# Septal myocardial scar burden predicts the response to cardiac contractility modulation in patients with heart failure

**DOI:** 10.1038/s41598-022-24461-6

**Published:** 2022-11-28

**Authors:** Uzair Ansari, Daniel Overhoff, Daniel Burkhoff, Christian Fastner, Gökhan Yücel, Susanne Röger, Boris Rudic, Volker Liebe, Martin Borggrefe, Ibrahim Akin, Jürgen Kuschyk, Theano Papavassiliu, Erol Tülümen

**Affiliations:** 1 First Department of Medicine, University Medical Center Mannheim, Faculty of Medicine Mannheim, University of Heidelberg, Theodor-Kutzer-Ufer 1-3, 68167 Mannheim, Germany; 2 Institute of Clinical Radiology and Nuclear Medicine, University Medical Center Mannheim, Medical Faculty Mannheim, Heidelberg University, Mannheim, Germany; 3 European Center for AngioScience (ECAS), Mannheim, Germany; 4 DZHK (German Center for Cardiovascular Research) Partner Site Heidelberg/Mannheim, Mannheim, Germany; 5 Department of Radiology, Bundeswehr Central Hospital Koblenz, Koblenz, Germany; 6 Cardiovascular Research Foundation, New York, NY USA; 7 Department of Cardiology, Faculty of Medicine, Koç University Hospital, Istanbul, Turkey

**Keywords:** Cardiology, Cardiac device therapy

## Abstract

We hypothesized that myocardial septal scarring, assessed by cardiac magnetic resonance (CMR) using late gadolinium enhancement (LGE), at the site of cardiac contractility modulation (CCM) lead placement may predict treatment response. Eligible heart failure (HF) patients underwent LGE CMR imaging before CCM device implantation. The response to CCM therapy at follow-up was determined by a change in NYHA class and echocardiographic left ventricular ejection fraction (LVEF) assessment. Patients were classified as responders, if they showed an improvement in either NYHA class or improvement of LVEF by ≥ 5%. 58 patients were included. 67% of patients were classified as responders according to improved NYHA; 55% according to LVEF improvement. 74% of patients were responders if either NYHA class or LVEF improvement was observed. 90% of responders (according to NYHA class) showed septal LGE < 25% at septal position of the leads, while 44% of non-responders showed septal LGE > 25% (p < 0.01). In patients treated with CCM, an improvement of NYHA class was observed when leads were placed at myocardial segments with a CMR- LGE burden less than 25%.

## Introduction

Heart failure (HF) continues to be a major cause of morbidity and mortality^1^. The use of electrical therapies such as cardiac resynchronization therapy (CRT) supplementing guideline directed medical therapy (GDMT) for patients with left bundle branch block or prolonged QRS has contributed to the management of severe HF. However, due to associated co-morbidities as well as gaps in treatment algorithms, the 5-year mortality rate has failed to improve significantly and remains high at 50%^2^.

Cardiac contractility modulation (CCM) offers a novel approach directed at patients with reduced left ventricular ejection fraction (LVEF) and normal QRS duration. This treatment involves the application of relatively high-voltage (~ 7.5 V) biphasic electric signals to the right ventricular (RV) septal wall, timed during the absolute refractory period of the myocardium for a duration of 20 ms^3^. These signals are not intended to initiate a new contraction or modify the activation sequence, rather it is assumed that CCM signals enhance contractile performance by effecting an increased release of calcium into the myocardial cells^4^. Randomized controlled trials as well as a recently concluded meta-analysis have suggested that CCM therapy improves symptoms, exercise tolerance and quality of life^5,6^. Additionally, data has also revealed a reduction in the 6-month composite rate of cardiac mortality and HF hospitalizations^7^.

The role of late gadolinium enhancement (LGE) in cardiac magnetic resonance (CMR) imaging for the stratification of risk in patients with cardiomyopathies has been assessed in a number of trials^8^. Additionally, the potential of CMR-LGE for predicting the risk and response in HF patients implanted with a cardiac device has also been well studied^9^. A study conducted by Taylor et al. could show that the deployment of a CRT left ventricular (LV) lead over non-scarred segments was associated with marked left ventricular remodeling and a better clinical outcome than a lead positioned over scarred segments^10^. As CCM leads are positioned at the right ventricular (RV) septum, we hypothesized that the presence or absence of scar at the septal myocardium may predict the response to therapy.

The aim of this study was to investigate the role of LGE derived by CMR as a predictor of response in HF patients implanted with a CCM device. We hypothesized that placement of CCM leads at non-scarred septal regions may result in a better clinical response by improving New York Heart Association (NYHA) class and/or LVEF.

## Materials and methods

### Study participants and design

This prospective, single-center, observational study included 58 consecutive HF patients implanted with a CCM Optimizer™ (Impulse Dynamics, Orangeburg, New York) device. All patients underwent contrast enhanced CMR before the procedure. Informed consent was obtained from all patients. This study was approved by our local ethics committee (Medizinische Ethikkommmision II, Medizinische Fakultät Mannheim) and was conducted according to standards of the Health Insurance Portability and Accountability Act (HIPAA) and the Declaration of Helsinki.

The study population consisted of patients above 18 years of age resistant to optimal medical therapy (OMT) for HF. Patients had a reduced LVEF (< 40%), as estimated by echocardiography and a QRS-duration of < 120 ms. All patients included in this study were implanted a CCM device and received an implantable cardioverter-defibrillator (ICD) if indicated. Patients with decompensated states of HF and reversible causes of HF such as valvular heart disease were excluded. Additionally, patients scheduled either for a coronary intervention or having undergone such a procedure during the last 3 months, patients with evidence of active ischemia, as well as those with a recent history of myocardial infarction (< 3 months) were excluded. The presence of a mechanical tricuspid valve, which could impede the placement of CCM leads was also cause for exclusion.

Patients were followed at 3-month intervals at our out-patient clinic. The Optimizer™ system was interrogated and a comprehensive clinical assessment including NYHA functional class and quality of life (using the Minnesota Living with Heart Failure Questionnaire, MLWHF), was conducted. Blood was sampled for the estimation of NT-proBNP levels. Echocardiography was performed for the determination of LVEF at baseline and at 1 year. Patients were classified as responders or non-responders to CCM therapy 1 year after implantation, based on an improvement in the NYHA functional class and/or LVEF.

### The CCM device

The patients recruited for this study were implanted with the Optimizer™ device system (bearing a CE mark), which included an implantable pulse generator (IPG), two right ventricular pacing leads, and an atrial sensing lead. A regular IS-1 bipolar pacemaker lead served as the atrial lead. Specific models of the Tendril lead (1888 T/2088 T by St. Jude Medical, Saint Paul, MN, USA) were used as ventricular leads. The Optimizer™ system delivers non-excitatory CCM signals to the heart 7 h per day. Each stimulus results in a non-excitatory, high amplitude (7.5 V), biphasic impulse of 20 ms duration applied to the RV septum during the absolute refractory period of the myocardium^11^.

**Figure 1:**
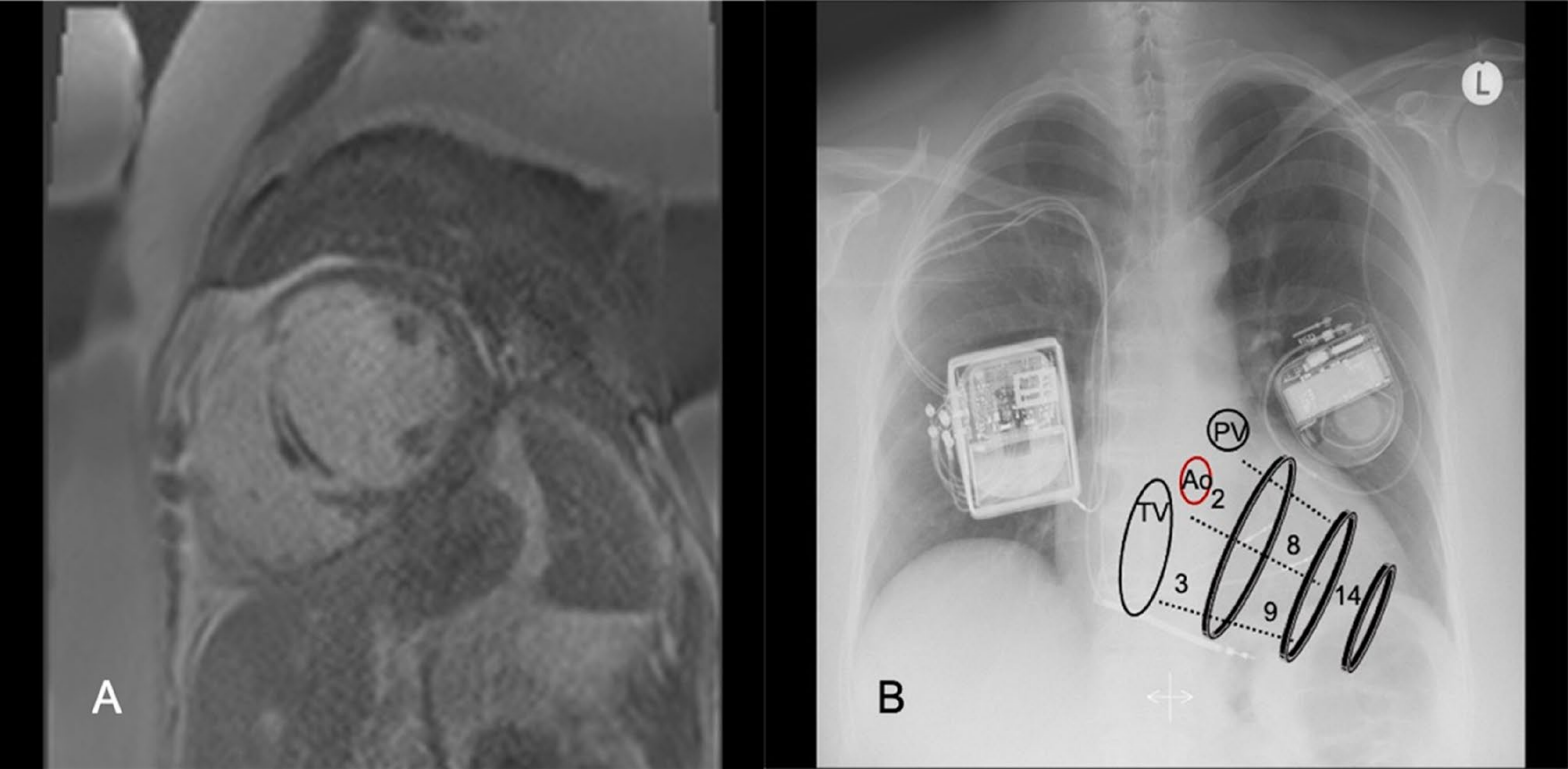
CCM implantation and placement of leads according to AHA segment model. (**A**) septal LGE in Segment 8 (LGE > 25%), (**B**) position of CCM leads at septum according to AHA-segment model.

The Optimizer™ CCM device was implanted under local anesthesia and conscious sedation. The placement of the ventricular bipolar screw-in leads in the right ventricular septum was done with fluoroscopic guidance and the septal position was confirmed using left and right anterior oblique views. A third lead (the atrial lead) was placed in the right atrium for sensing purposes. One ventricular lead was placed at the anterior septal groove and the other lead in the posterior groove, approximately halfway between the base and apex of the right ventricle. The location of lead tip was determined after evaluation of operative data (LAO 60 and RAO 30) and post-operative postero-anterior and lateral chest radiographs by a trained radiologist. This region was subsequently labelled to correspond to the American Heart Association (AHA) tomographic imaging segmentation model^12^ (Fig. 1).

### Image acquisition and analysis

The acquisition and analysis of echocardiographic and CMR images has been described in detail in the supplements.

### Outcome measures

Patients were classified as responders, if they showed an improvement in either NYHA class or improvement of LVEF by ≥ 5%. Patients were classified as non-responders if symptoms persisted/worsened, or if the LVEF did not show a change. Other clinical endpoints were cardiovascular mortality, the composite of death from any cause and an unplanned hospitalization for major adverse cardiovascular events (MACE), re-hospitalization for worsening heart failure, myocardial infarction, acute coronary syndromes, stroke, or pulmonary embolism.

### Analysis of LGE at position of lead tip(s)

A dual lead CCM device could also be programmed to function with a single active lead, continuing to ensure optimal CCM action. In a head-to-head comparison between CCM systems with dual lead energy delivery and single active lead energy delivery it is imperative to consider the position of both lead tips on the myocardial segment. For this purpose, we further categorized patients into two sub-groups. Group A included patients with single active lead as well as patients with both leads from the dual lead system, which were all positioned at scar (LGE > 25%). Group B included patients with a single active lead, where tips were not positioned at scar, or when they had a dual lead system, at least one lead was not positioned at scar (LGE < 25%).

### Statistical analysis

Continuous variables are expressed as mean ± SD (standard deviation) in case of normal distribution and as the median and interquartile range when the distributions were skewed. The differences in mean values between 2 groups were analyzed with Student’s t-test for independent samples. When distribution of variable is skewed, the Mann–Whitney U-test was used to analyse the differences between 2 groups.

Comparison between continuous variables was performed with Mann–Whitney U test and between categorical variables with Pearson Chi-Square test with continuity correction, Fisher’s exact test or Mann–Whitney U test. A p-value of < 0.05 was considered statistically significant and all tests were 2-tailed.

All statistical analysis was performed using Statistical Package for the Social Sciences (IBM SPSS Statistics, version 20.0 for Macintosh; SPSS, Inc., Chicago, IL, USA).

## Results

A total of 58 HF patients implanted with a CCM Optimizer™ device underwent CMR before the procedure.

### Baseline characteristics of total population

The study cohort was predominantly male with a median age of 57 years (28–81 years, 86.2% male). Majority of patients included in this study had a reduced ejection fraction (LVEF < 35%, 93.1%) and the mean LVEF was 24.4% (range, 12–37%). The mean change in LVEF after implantation was + 4.44%. All patients were implanted with an ICD. Patients exhibited varying grades of symptoms and 75.9% of the study population were in NYHA III. The etiology of the underlying HF in the study cohort was either ischemic (46.6%) or non-ischemic (53.4%). Most patients received angiotensin-converting enzyme inhibitors and diuretics (94.8% and 93.1%, respectively) unless contraindications precluded their use. 96.6% of the patients received betablockers and 65.5% of the patients received aldosterone inhibitors. There was no mortality reported in the group during the follow-up period. The CMR analysis showed that septal LGE was present in 72.4% of all study patients (Table 1).

**Table 1:** Baseline characteristics.

Variable	Total population (n = 58)
**Gender—male, n (%)/female, n (%)**	50 (86.2%)/8 (13.8%)
**Age [years], median (IQR)**	57 (28; 81)
**Body mass index [kg/m**^**2**^**], median (IQR)**	28.40 (16; 44)
LVEF, mean [%]; median [%] (IQR) (echocardiographic estimate)	24.4%; 25% (12; 37)
**NYHA class at baseline**	
NYHA I	1 (1.7%)
NYHA II	10 (17.2%)
NYHA III	44 (75.9%)
NYHA IV	3 (5.2%)
**Aetiology of HF—ischemic, n (%)/non-ischemic, n (%)**	27 (46.6%)/31 (53.4%)
**ICD, n (%)**	58 (100%)
Primary indication	54 (93.1%)
Secondary indication	4 (6.9%)
**Arterial hypertension, n (%)**	38 (65.5%)
**Diabetes mellitus, n (%)**	20 (34.5%)
**Chronic kidney disease, n (%)**	28 (48.3%)
ACE inhibitors/AT-1 blockers	55 (94.8%)
Diuretics	54 (93.1%)
Betablockers	56 (96.6%)
Aldosterone inhibitors	38 (65.5%)
Ivabradine	6 (10.3%)
Entresto	2 (3.4%)
**Optimizer—lead stimulation, n (%)**	
Single stimulation	18 (31%)
Dual stimulation	40 (69%)
**CMR—septal LGE, n (%)**	42 (72.4%)

*LVEF* left ventricular ejection fraction, *NYHA* New York Heart Association, *HF* heart failure, *ICD* implantable cardioverter-defibrillator, *COPD* Chronic obstructive pulmonary disease, *ACE* angiotensin converting enzyme, *AT-1* angiotensin receptor, *CMR* cardiac magnetic resonance, *LGE* late gadolinium enhancement.

### Comparison between responders and non-responders

#### Responders vs. non-responders according to NYHA change

A total of 67.2% of all patients were classified as responders based on the change in NYHA class. There was no significant difference between the responders and non-responders regarding baseline demographic, OMT, and imaging parameters as outlined in Supplementary Table 1. The amount of septal LGE revealed a trend (p = 0.06), with responders showing lesser scar tissue as compared to non-responders.

Table 2 shows a general trend to improvement in the MLWHF score (p = 0.06) as well as significant decrease in NT-proBNP levels in patients showing response to CCM therapy (P < 0.01). 76.9% of all responders reported an improvement of one NYHA class on follow-up and the mean change in LVEF was 6.0% (p < 0.01 for both variables). There was a pronounced improvement in longitudinal right ventricular (RV) function (as measured by TAPSE) amongst the responders (p = 0.06), but although this relationship revealed a trend, it was not statistically significant.

**Table 2:** Responder vs. non-responder based on NYHA change.

Variable	NYHA change			
		Responder (n = 39)	Non-responder (n = 19)	p-value*
LVEF change [%]		6.0 ± 6.7	1.3 ± 3.1	** < 0.01**
TAPSE change [mm]		1.5 ± 2.3	0.3 ± 1.7	0.06
MLWHF change		− 19.3 ± 17.4	-5.6 ± 16.4	0.06
NT-pro BNP (pg/mL) change		− 1912 ± 2490	1431 ± 5178	** < 0.01**
NYHA change, n (%)	− 2	9 (23.1%)	–	** < 0.01**
	− 1	30 (76.9%)	–	
	0	–	17 (89.5%)	
	+ 1	–	2 (10.5%)	
Lead stimulation, n (%)	Single	12 (30.8%)	6 (31.6%)	0.95
	Dual	27 (69.2%)	13 (68.4%)	
LGE of at least 25% at lead position (both single and dual)	LGE > 25% (Group A)	4 (10.3%)	6 (44.4%)	** < 0.01**
	LGE < 25% (Group B)	35 (89.7%)	10 (55.6%)	

*LGE* late gadolinium enhancement, *NYHA* New York Heart Association, *FU* follow-up.

Values in bold are statistically significant.

*Pearson Chi-square test.

CCM energy delivery through either single (30.8% vs. 31.6%) or two leads (69.2% vs. 68.4%) did not reveal any differences between the responders and non-responders (p = NS). Patients classified as responders according to change in NYHA class showed a significant difference to non-responders according to amount of LGE (25%) at the septal lead position. Responders primarily comprised of patients from Group B (89.7%), whereas almost half of the non-responders comprised of patients from Group A (44.4%) (p < 0.01) (Table 2).

#### Responders vs. non-responders according to LVEF change

Around 44.8% of all patients were classified as responders based on the change in LVEF. There was no significant difference between the responders and non-responders regarding baseline demographic, OMT, and imaging parameters as outlined in Supplementary Table 1.

Table 3 shows a more prominent decrease in NT-proBNP levels in patients showing response to CCM therapy (p = NS). 84.6% of patients from the responder group showed improvement of the NYHA class. Interestingly 53.2% of the non-responders also showed improvement in NYHA (p = 0.04). The mean change in LVEF was 9.2% in the responder group, while the mean TAPSE change was 2.1 mm, both of which were significant (p < 0.01).

**Table 3:** Responder vs. non-responder based on LVEF change.

Variable	LVEF change			
		Responder (n = 26)	Non-responder (n = 32)	p-value*
LVEF change [%]		9.2 ± 6.3	0.4 ± 1.2	** < 0.01**
TAPSE change [mm]		2.1 ± 2.4	0.2 ± 1.4	** < 0.01**
MLWHF change		− 11.3 ± 16.5	− 19.5 ± 18.6	0.20
NT-pro BNP (pg/mL) change		− 1995 ± 2921	68.4 ± 4170	0.06
NYHA change, n (%)	− 2	6 (23.1%)	3 (9.4%)	**0.04**
	− 1	16 (61.5%)	14 (43.8%)	
	0	4 (15.4%)	13 (40.6%)	
	+ 1	–	2 (6.3%)	
Lead stimulation, n (%)	Single	8 (30.8%)	10 (31.3%)	0.96
	Dual	18 (69.2%)	22 (68.8%)	
LGE of at least 25% at lead position (both single and dual)	LGE > 25% (Group A)	4 (15.4%)	8 (25.8%)	0.33
	LGE < 25% (Group B)	22 (84.6%)	23 (74.2%)	

*LGE* late gadolinium enhancement, *NYHA* New York Heart Association, *FU* follow-up.

Values in bold are statistically significant.

*Pearson Chi-square test.

The CCM energy delivery through either single (30.8% vs. 31.2%) or two leads (69.2% vs. 68.8%) did not reveal any difference in the effectiveness of CCM therapy between the responders and non-responders (p = NS). Interestingly, patients classified as responders according to change in LVEF did not show a significant difference to non-responders according to amount of LGE (25%) at the septal lead position. Responders primarily comprised of patients from Group B (84.6%), whereas 25.8% of the non-responders comprised of patients from Group A (p = NS) (Table 3).

#### Responders vs. non-responders according to improvement of NYHA or LVEF

Improvement of NYHA and/or LVEF change were observed in 74.1% of patients. There were no significant differences between responders and non-responders regarding baseline demographic, OMT, baseline demographic and imaging parameters as outlined in Supplementary Table 1.

Supplementary Table 2 shows that the stimulation through either single (32.6% vs. 26.7%) or dual leads (67.4% vs. 73.3%) did not reveal any differences in the response to CCM therapy (p = NS). Patients classified according to the amount of LGE (25%) showed a significant difference in response (p < 0.01). Responders primarily comprised of patients from Group B (86%), whereas almost half of the non-responders comprised of patients from Group A (42.9%) (p = 0.02) (Supplementary Table 2).

## Discussion

This study investigated the role of LGE in predicting response to CCM therapy in HF patients based on lead placement at the septal wall. The results revealed that significantly greater number of HF patients responded to CCM therapy, showing either an improvement in NYHA class or LVEF when leads were positioned over minimally scarred or non-scarred regions of the septal myocardium.

The importance of precise positioning of cardiac device leads in electrical therapies for HF has been explored in a few CRT trials. Rademakers et al., used canine LV free-wall infarct models to study optimal CRT response and suggested that pacing outside of scarred infarct zones could show a clinical benefit^13^. In one clinical trial conducted by Shajil et al., a transmural scar predicted a negative response among ICM patients treated with a CRT device^14^. This concurred with results from echocardiographic studies in ICM patients by Adelstein et al., wherein greater scar density near the LV lead tip, as demonstrated by myocardial perfusion imaging, was associated with an unfavorable response to CRT^15^.

There are, however, fundamental differences in the physiological actions of CCM and CRT. CCM signals are unique and differ from pacing pulses, as they influence cardiac inotropy by improving the function of cardiac myocytes rather than initiate a new contraction^16^. It is believed that signals delivered by a CCM device influence myocardial properties by inducing significant changes in myocardial gene expression, improved expression and phosphorylation of the sodium-calcium exchanger, phospholamban and connexin 43, upregulation of SERCA-2A, as well as reverse fetal gene programming observed in HF^3^. Nevertheless, it is believed that the favorable genomic remodeling occurring through CCM, requires the lead to be placed at viable septal myocardium (as opposed to RV free wall), thus affording best signal delivery to the LV^2^.

In the context of our CCM study, delivery of stimulus was associated with an effective response, wherein patients reported an improvement in NYHA class when CCM leads (both single and dual) were placed at septal segments with a LGE burden lesser than 25%. The prognostic use of CMR LGE quantified myocardial scar among CRT patients was explored in a study by White et al., which could conclude that a percent septal scar > 40% accurately identified patients reporting poor clinical response^17^. In another CRT trial comprising 559 HF patients, LGE in the myocardial segment subtended by the LV lead tip was associated with a poorer response and negative outcomes, thus leading the authors to recommend the pre-operative use of CMR-LGE to guide LV lead placement^18^. Our review of CCM literature yielded no data exploring the use of CMR LGE for guiding CCM lead placement.

A recent study by Anker et al. conducted in patients with LVEF between 25 and 45%, reported a better clinical response to CCM therapy among those with higher LVEF^19^. This finding has been consistently reported in earlier studies, but the underlying mechanism dictating such a response has not yet been defined. It has been hypothesized that patients with lower EF are more likely to have more scar and less muscle mass, thereby influencing the benefits of CCM therapy^7^. Our results potentially support this hypothesis by suggesting that low or negligible septal myocardial scar as quantified by LGE predicts an improved response to CCM therapy. A similar observation was also reported in a CRT trial conducted by Duckett et al., wherein a trend towards greater ventricular remodeling and better acute hemodynamic response was reported when leads were placed at regions without LGE^20^.

Our analysis also revealed an improved clinical response to CCM therapy using two leads, even if one of the leads was placed at septal myocardium with a LGE burden > 25%. This response was comparable to the use of a single lead CCM, extrapolating the conclusion from an earlier study conducted at our center, which suggested that CCM signals delivered using one ventricular lead were no less efficacious than when delivered through two ventricular leads simultaneously^11^.

For purposes of this study, additional analyses were performed with patients classed as responders if they showed an improvement in either LVEF or NYHA class. This analysis also corroborated our earlier findings by suggesting that positioning of leads over septal myocardial scarring greater than 25% was associated with a poorer response.

Another observation in our analysis was the absence of any significant difference between responders and non-responders based on total myocardial scarring (LGE). Interestingly, an early CRT study conducted by Harb et al., reported non-responders having significantly higher levels of total scar as compared to CRT-responders, and identified an increase in scar burden to be associated with worse outcomes^21^. The investigators hypothesized that the presence of scar in any wall impeded global LV remodeling, thereby limiting the response to resynchronization therapy. As CCM also influences global LV remodeling it is interesting to note the difference in response between effects of CCM signals and CRT pacing at septal myocardium.

The lack of corroborative data in our study makes it difficult to automatically assume the positive impact of CCM when leads are placed in septal myocardial segments with reduced scarring as seen in multiple CRT studies. However, the improvement in NYHA functional class and LVEF does suggest that these patients could benefit from a CMR to identify myocardial scarring before device implantation. Considering the potential for wider adoption of CCM in HF patients, it is therefore prudent to further investigate its role using CMR as a guide for lead placement in larger RCTs.

## Study limitations

We used analyses from CRT trials as comparison to study the predictive role of LGE, considering the use of similar leads and the physiological basis of signal delivery to myocardium. Another limitation was the small patient cohort, which limited a detailed sub-classification of patients according to LGE. The use of echocardiography at a single-point follow-up after 1 year could also be considered a limiting factor. The cut-off 5% in LVEF change, leaves room for discrepancy considering the significant test–retest as well as inter-reader variability.

## Conclusions

The results of this study show that septal myocardial scar burden at the lead position assessed by CMR-LGE could help in predicting response to CCM therapy. An improvement in the NYHA class was reported at follow-up when leads were placed at segments with a scar burden lesser than 25%. This relationship also persisted even when a change in either NYHA class or LVEF was used as a measure of outcome.

## Supplementary Information

Supplementary Information.

## Data Availability

All data used during the current study are available from the corresponding author upon request.
