# Supplementary material for: Septal myocardial scar burden predicts the response to cardiac contractility modulation in patients with heart failure
Source: Sci Rep. 2022 Nov 28;12:20504. doi: 10.1038/s41598-022-24461-6 (PMC9705404; doi:10.1038/s41598-022-24461-6)
Supplement: Supplementary file 1 — Supplementary Information. [file 41598_2022_24461_MOESM1_ESM.docx]

**Supplement Text**

***CMR image acquisition***

All CMR studies were performed using a 1.5 Tesla or a 3 Tesla whole-body imaging system (MAGNETOM SONATA, AVANTO and SKYRA (3T), Siemens Medical System, Healthcare sector, Erlangen, Germany) using a 4-element (Sonata), a 6-element (Avanto) phased-array body coil and an 18-element body matrix coil (Skyra). Cine images were acquired using a retrospective ECG-gated, balanced segmented steady state free precession (trueFISP) sequence in 3 long-axis views (2-, 3-, and 4-chamber view) and in multiple short-axis views, covering the entire LV from base to apex. Body weight adapted late gadolinium enhancement (LGE) images of the standard axis were acquired 10-15 minutes after i.v. injection of 0.2 mmol/kg Gadoteric acid (Dotarem, Guerbet, Roissy CdG Cedex, France, Germany), using an inversion recovery turbo fast low angle shot sequence at the same position as the long- and short- axis cine acquisitions in end-diastole. The myocardial signal was “nulled” by adjusting inversion recovery (IR) time individually for every patient, typically resulting in an IR time between 240 and 300ms.

***CMR image analysis***

The analysis of CMR DICOM images was performed using *cvi42* software (Circle Cardiovascular Imaging Inc., Calgary, Canada). LVEF, volumes and mass were quantified from the cine images by standard methods and these parameters were then normalized to body surface area. The myocardium was divided into 16 segments, adapted from the American Heart Association (AHA) tomographic imaging model. [11] CMR images were analyzed for the presence and extent of LGE by at least two certified CMR specialists (radiologist & cardiologist) blinded to clinical outcome. Each segment was scored on a self-derived scale by visually estimating the extent of LGE. Segments with no visible delayed enhancement were scored as 0. The presence of LGE involving less than 25% of the segment myocardium was scored as 1; 26 - 50% as 2; 51 - 75% as 3; and a transmural scar (>75%) as 4. The global extent of LGE was calculated summing all segmental scores. The volume of LGE in LV septum was expressed as a percentage after estimation of scar in the septal segments (AHA model 2, 3, 8, 9, and 14).

***Echocardiography analysis***

Transthoracic echocardiography was performed in all patients along with CMR using different models of a phased-array ultrasound sector scanner (Vivid, GE Healthcare, UK) and a 3.5-MHz transducer. A follow-up echocardiography was done after 1 year. Patients were positioned in supine / left lateral decubitus manner, and 2D images and measurements were obtained in the standard parasternal and apical views. Doppler recordings were recorded using a sweep speed of 100 m/s.

**Supplementary Table 1. Baseline demographic and imaging parameters according to response determinants**

| **Variable** | **NYHA** | | | **LVEF** | | | **NYHA or LVEF** | | |
| --- | --- | --- | --- | --- | --- | --- | --- | --- | --- |
|  | **Responder**  **(n = 39)** | **Non-Responder**  **(n = 19)** | **p-value** | **Responder**  **(n = 32)** | **Non-Responder**  **(n = 26)** | **p-value** | **Responder**  **(n = 43)** | **Non-responder**  **(n = 15)** | **p-value** |
| *Cohort (%)* | 67.2% | 32.8% | - | 55.2% | 44.8% | - | 74.1% | 25.9% | - |
| *ACEI/ AT1*  *Beta-blockers*  *Diuretics*  *Aldoster. Inh* | 37 (94.9%)  38 (97.4%)  37 (94.9%)  27 (69.2%) | 18 (94.7%)  18 (94.7%)  17 (89.5%)  11 (57.9%) | NS | 25 (96.2%)  25 (96.2%)  24 (92.3%)  20 (76.9%) | 30 (93.8%)  31 (96.9%)  30 (93.8%)  18 (56.3%) | NS | 40 (93.0%)  42 (97.7%)  40 (93.0%)  31 (72.1%) | 15 (100%)  14 (93.3%)  14 (93.3%)  7 (46.7%) | NS |
| *Ischemic*  *vs.*  *non-ischemic CM* | 20 (51.3%) | 7 (36.8%) | NS | 10 (38.5%) | 17 (53.1%) | NS | 22 (51.2%) | 5 (33.3%) | NS |
|  | 19 (48.7%) | 12 (63.2%) |  | 16 (61.5%) | 15 (46.9%) |  | 21 (48.8%) | 10 (66.7%) |  |
| **Echocardiography parameters** | | | | | | | | | |
| *LVEF (%)* | 24.2 ± 6.6 | 25 ± 6.4 | 0.64 | 23.5 ± 6.4 | 25.2 ± 6.5 | 0.35 | 24.37 ± 6.3 | 24.6 ± 7.1 | 0.9 |
| *TAPSE* | 16.5 ± 3.8 | 17.4 ± 4.4 | 0.44 | 16.6 ± 3.4 | 17.0 ± 4.4 | 0.69 | 16.5 ± 3.7 | 17.6 ± 4.7 | 0.36 |
| **CMR parameters** | | | | | | | | | |
| *LVEF (%)* | 26.0 ± 9.5 | 25.5 ± 7.0 | NS | 24.7 ± 8.9 | 26.9 ± 8.5 | NS | 25.9 ± 9.2 | 25.7 ± 7.5 | NS |
| *SWT (mm)* | 9.5 ± 2.8 | 9.8 ± 2.0 |  | 9.1 ± 2.9 | 10.0 ± 2.2 |  | 9.4 ± 2.7 | 10.0 ± 2.0 |  |
| *PWT (mm)* | 7.9 ± 2.6 | 8.2 ± 2.0 |  | 7.8 ± 2.4 | 8.1 ± 2.4 |  | 7.8 ± 2.5 | 8.3 ± 2.1 |  |
| *RVEF (%)* | 45.8 ± 15.1 | 47.6 ± 16.1 |  | 45.5±14.9 | 47.1±15.9 |  | 46.2 ± 15.1 | 46.9 ± 16.5 |  |
| *Septal LGE (%) of myocardium* | 20.7 ± 19.3 | 33.2 ± 27.0 |  | 21.4±20.1 | 27.2±24.3 |  | 22.5 ± 20.3 | 31.1±28.4 |  |
| *Total LGE, %* | 22.9±17.2 | 25.0±20.1 |  | 23.7±19.2 | 23.6±17.4 |  | 24.5±18.1 | 21.2±18.4 |  |
| *LVEF – left ventricular ejection fraction, CM- Cardiomyopathy, TAPSE – tricuspid annular plane systolic excursion, LVEDD – left ventricular end-diastolic diameter, SWT – septal wall thickness, PWT – posterior wall thickness, RVEF - right ventricular ejection fraction, RVEDD – right ventricular end-diastolic diameter, LGE – late gadolinium enhancement, MLWHF – Minnesota Living with Heart Failure, NT-proBNP – N-terminal pro- Brain natriuretic peptide, FU – follow-up, SD – Standard deviation, NS – Not significant*  ***Values in bold are statistically significant*** | | | | | | | | | |

**Supplementary Table 2. Responder vs. Non-responder based on either NYHA or LVEF change**

| **Variable** | **NYHA or LVEF change** | | | |
| --- | --- | --- | --- | --- |
|  |  | **Responder**  **(n = 43)** | **Non-Responder**  **(n = 15)** | **p-value*** |
| **Lead stimulation, n (%)** | *Single* | 14 (32.6%) | 4 (26.7%) | 0.67 |
|  | *Dual* | 29 (67.4%) | 11 (73.3%) |  |
| **LGE of at least 25% at lead position (both single and dual)** | *LGE > 25%*  *(Group A)* | 6 (14%) | 6 (42.9%) | **0.02** |
|  | *LGE < 25%*  *(Group B)* | 37 (86%) | 8 (57.1%) |  |
| *Pearson Chi-square test  LGE – late gadolinium enhancement, NYHA – New York Heart Association, FU – Follow-up  ***Values in bold are statistically significant*** | | | | |
